# Crystal structure of ethyl 2-(1*H*-benzimidazol-2-yl)-2-[2-(4-nitro­phen­yl)hydrazinyl­idene]acetate

**DOI:** 10.1107/S2056989015004818

**Published:** 2015-03-14

**Authors:** Mohamed Loughzail, Abdesselam Baouid, Lahcen El Ammari, Mohamed Saadi, Moha Berraho

**Affiliations:** a Laboratoire de Chimie Moléculaire, Faculté des Sciences Semlalia, BP 2390, Université Cadi Ayyad, 40001 Marrakech, Morocco; b Laboratoire de Chimie du Solide Appliqué, Faculté des Sciences, Avenue Ibn Battouta, BP 1014 Rabat, Morocco; c Laboratoire de Chimie des Substances Naturelles, URAC16, Faculté des Sciences Semlalia, BP 2390 Bd My Abdellah, 40000 Marrakech, Morocco

**Keywords:** crystal structure, benzimidazole, nitro­phenyl­hydrazone, hydrogen bonding

## Abstract

The title compound, C_17_H_15_N_5_O_4_, was obtained *via* the condensation of 3-eth­oxy-2-[2-(4-nitro­phen­yl)hydrazono]-3-oxo­propanoic acid with 1,2-di­amino­benzene. In the mol­ecule, the dihedral angles between the acetate group and the two aromatic subunits (benzimidazole and nitro­phenyl­hydrazone) are 7.35 (9) and 18.23 (9)°, respectively. Intra­molecular N—H⋯O and N—H⋯N contacts occur. In the crystal, C—H⋯O and N—H⋯O hydrogen bonds link the mol­ecules into chains along the *b*-axis direction.

## Related literature   

For the pharmacological activity of benzimidazole derivatives, see: Luo *et al.* (2011 ▸); Ouattara *et al.* (2011 ▸); Bhrigu *et al.* (2012 ▸); Singh *et al.* (2012 ▸); Parajuli *et al.* (2014 ▸). For their agrochemical activity, see: Attrassi *et al.* (2007 ▸).
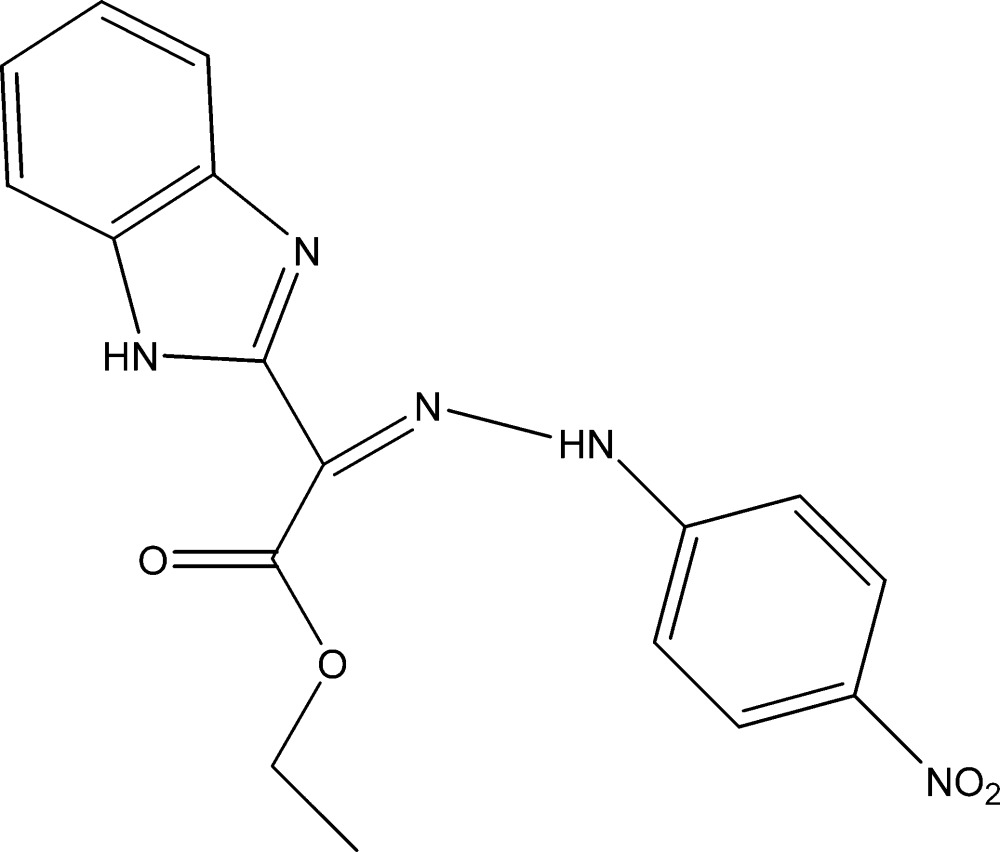


## Experimental   

### Crystal data  

C_17_H_15_N_5_O_4_
*M* *_r_* = 353.34
Monoclinic, 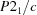

*a* = 12.877 (5) Å
*b* = 5.874 (5) Å
*c* = 21.988 (5) Å
β = 99.060 (5)°
*V* = 1642.4 (16) Å^3^
*Z* = 4
Mo *K*α radiation
μ = 0.11 mm^−1^
*T* = 293 K
0.33 × 0.17 × 0.04 mm

### Data collection  

Bruker APEXII CCD diffractometer
24995 measured reflections
3362 independent reflections
2562 reflections with *I* > 2σ(*I*)
*R*_int_ = 0.033

### Refinement  

*R*[*F* ^2^ > 2σ(*F* ^2^)] = 0.038
*wR*(*F* ^2^) = 0.108
*S* = 1.03
3362 reflections
236 parameters
H-atom parameters constrained
Δρ_max_ = 0.19 e Å^−3^
Δρ_min_ = −0.17 e Å^−3^

### 

Data collection: *APEX2* (Bruker, 2009 ▸); cell refinement: *SAINT-Plus* (Bruker, 2009 ▸); data reduction: *SAINT-Plus*; program(s) used to solve structure: *SHELXS97* (Sheldrick,2008 ▸); program(s) used to refine structure: *SHELXL97* (Sheldrick,2008 ▸); molecular graphics: *ORTEP-3 for Windows* (Farrugia, 2012 ▸); software used to prepare material for publication: *WinGX* (Farrugia, 2012 ▸).

## Supplementary Material

Crystal structure: contains datablock(s) I. DOI: 10.1107/S2056989015004818/im2461sup1.cif

Structure factors: contains datablock(s) I. DOI: 10.1107/S2056989015004818/im2461Isup2.hkl

Supporting information file. DOI: 10.1107/S2056989015004818/im2461Isup3.cml

**Fig. 1.**
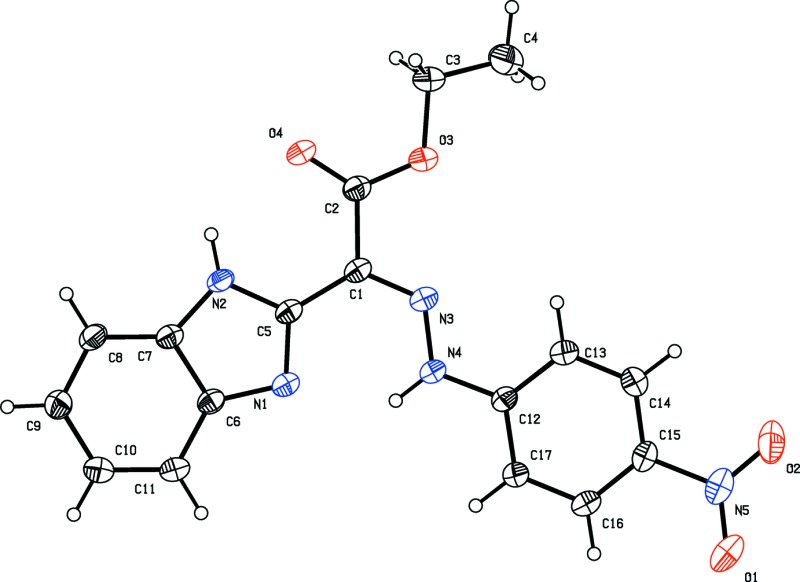
: Molecular structure of the title compound with displacement ellipsoids drawn at the 30% probability level. H atoms are represented as small spheres of arbitrary radii.

**Fig. 2.**
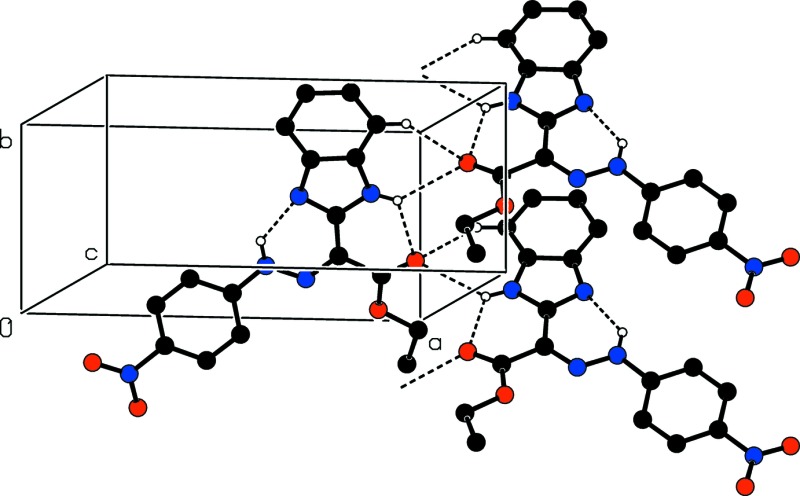
: Partial packing view showing the C—H···O and N—H···O interactions (dashed lines) and the formation of a chain parallel to the b axis. H atoms not involved in hydrogen bonding have been omitted for clarity.

. DOI: 10.1107/S2056989015004818/im2461fig1.tif
Mol­ecular structure of the title compound with displacement ellipsoids drawn at the 30% probability level. H atoms are represented as small spheres of arbitrary radii.

b . DOI: 10.1107/S2056989015004818/im2461fig2.tif
Partial packing view showing the C—H⋯O and N—H⋯O inter­actions (dashed lines) and the formation of a chain parallel to the *b* axis. H atoms not involved in hydrogen bonding have been omitted for clarity.

CCDC reference: 1052904

Additional supporting information:  crystallographic information; 3D view; checkCIF report

## Figures and Tables

**Table 1:** Hydrogen-bond geometry (, )

*D*H*A*	*D*H	H*A*	*D* *A*	*D*H*A*
N2H2O4^i^	0.86	2.50	3.161(3)	134
C8H8O4^i^	0.93	2.54	3.258(3)	134
N2H2O4	0.86	2.21	2.750(3)	121
N4H4N1	0.86	2.02	2.679(3)	133

Symmetry code: (i) 
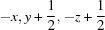
.

## supplementary crystallographic information

### S1. Comment

The development of an efficient synthesis of bioactive compounds is a major challenge in modern chemistry. The high therapeutic properties of benzimidazole derivatives related drugs have encouraged the medicinal chemists to synthesize a large number of new chemotherapeutic agents. The benzimidazole motif is an integral part in numerous fields, as pharmaceuticals (Luo *et al.*, 2011; Ouattara *et al.*, 2011; Bhrigu *et al.*, 2012; Singh *et al.*, 2012; Parajuli *et al.*, 2014), and agrochemicals (Attrassi *et al.*, 2007). The structure of this new product was determined by its single-crystal X-ray structure. The dihedral angles between the acetate chain and the two aromatic subunits (benzimidazole and nitrophenylhydrazone) are 7.35 (9)° and 18.23 (9)°, respectively. In the crystal structure, the molecules are linked by C—H···O and N—H···O intermolecular hydrogen bonds into chains along the *b* axis (Fig.2). In addition an intramolecular N—H···O hydrogen bond is also observed.

### S2. Experimental

1,2-diaminobenzene (0.5 g, 4.6 mmol) and 3-ethoxy-2-[2-(4-nitrophenyl)hydrazono]-3-oxopropanoic acid (1.19 g, 4.6 mmol) were heated in xylene (15 ml) for 12 h. The solvent was evaporated. The title compound was isolated by column chromatography on silica gel using hexane/ethyl acetate as eluent. The solid product was recrystallized in dichloromethane at 15°C to give yellow crystals (yield: 45%) of the title compound.

### S3. Refinement

All H atoms were fixed geometrically and treated as riding with C—H = 0.96 Å (methyl), 0.97 Å (methylene) and 0.98 Å (methine) with *U*_iso_(H) = 1.2 Ueq(methylene, methine and OH) or *U*_iso_(H) = 1.5 Ueq(methyl).

### Crystal data

**Table:**

C_17_H_15_N_5_O_4_	*F*(000) = 736
*M**_r_* = 353.34	*D*_x_ = 1.429 Mg m^−^^3^
Monoclinic, *P*2_1_/*c*	Mo *K*α radiation, λ = 0.71073 Å
Hall symbol: -P 2ybc	Cell parameters from 3362 reflections
*a* = 12.877 (5) Å	θ = 2.7–26.4°
*b* = 5.874 (5) Å	µ = 0.11 mm^−^^1^
*c* = 21.988 (5) Å	*T* = 293 K
β = 99.060 (5)°	Platelet, colourless
*V* = 1642.4 (16) Å^3^	0.33 × 0.17 × 0.04 mm
*Z* = 4	

### Data collection

**Table:**

Bruker APEXII CCD diffractometer	2562 reflections with *I* > 2σ(*I*)
Radiation source: fine-focus sealed tube	*R*_int_ = 0.033
Graphite monochromator	θ_max_ = 26.4°, θ_min_ = 2.7°
ω and φ scans	*h* = −16→16
24995 measured reflections	*k* = −7→7
3362 independent reflections	*l* = −27→27

### Refinement

**Table:**

Refinement on *F*^2^	Primary atom site location: structure-invariant direct methods
Least-squares matrix: full	Secondary atom site location: difference Fourier map
*R*[*F*^2^ > 2σ(*F*^2^)] = 0.038	Hydrogen site location: inferred from neighbouring sites
*wR*(*F*^2^) = 0.108	H-atom parameters constrained
*S* = 1.03	*w* = 1/[σ^2^(*F*_o_^2^) + (0.0471*P*)^2^ + 0.5028*P*] where *P* = (*F*_o_^2^ + 2*F*_c_^2^)/3
3362 reflections	(Δ/σ)_max_ < 0.001
236 parameters	Δρ_max_ = 0.19 e Å^−^^3^
0 restraints	Δρ_min_ = −0.17 e Å^−^^3^

### Special details

**Table:**

Geometry. All s.u.'s (except the s.u. in the dihedral angle between two l.s. planes) are estimated using the full covariance matrix. The cell s.u.'s are taken into account individually in the estimation of s.u.'s in distances, angles and torsion angles; correlations between s.u.'s in cell parameters are only used when they are defined by crystal symmetry. An approximate (isotropic) treatment of cell s.u.'s is used for estimating s.u.'s involving l.s. planes.
Refinement. Refinement of *F*^2^ against ALL reflections. The weighted *R*-factor *wR* and goodness of fit *S* are based on *F*^2^, conventional *R*-factors *R* are based on *F*, with *F* set to zero for negative *F*^2^. The threshold expression of *F*^2^ > 2σ(*F*^2^) is used only for calculating *R*-factors(gt) *etc*. and is not relevant to the choice of reflections for refinement. *R*-factors based on *F*^2^ are statistically about twice as large as those based on *F*, and *R*- factors based on ALL data will be even larger.

### Fractional atomic coordinates and isotropic or equivalent isotropic displacement parameters (Å^2^)

**Table:**

	*x*	*y*	*z*	*U*_iso_*/*U*_eq_	
C1	0.24083 (11)	0.7913 (3)	0.32924 (7)	0.0367 (3)	
C12	0.49273 (11)	0.6017 (3)	0.39833 (6)	0.0349 (3)	
C13	0.46733 (12)	0.3904 (3)	0.42102 (7)	0.0405 (4)	
H13	0.3980	0.3398	0.4147	0.049*	
C5	0.25493 (11)	0.9962 (3)	0.29411 (7)	0.0357 (3)	
C15	0.64834 (12)	0.3339 (3)	0.46186 (7)	0.0415 (4)	
C2	0.13384 (12)	0.6936 (3)	0.32829 (7)	0.0413 (4)	
C17	0.59698 (12)	0.6755 (3)	0.40730 (7)	0.0406 (4)	
H17	0.6138	0.8155	0.3917	0.049*	
C6	0.32726 (11)	1.2789 (3)	0.25402 (7)	0.0365 (3)	
C11	0.39466 (12)	1.4456 (3)	0.23679 (7)	0.0410 (4)	
H11	0.4662	1.4438	0.2521	0.049*	
C16	0.67524 (12)	0.5414 (3)	0.43928 (7)	0.0439 (4)	
H16	0.7448	0.5900	0.4455	0.053*	
C8	0.17657 (12)	1.4543 (3)	0.18916 (8)	0.0460 (4)	
H8	0.1052	1.4564	0.1733	0.055*	
C9	0.24422 (13)	1.6167 (3)	0.17267 (8)	0.0470 (4)	
H9	0.2180	1.7311	0.1453	0.056*	
C14	0.54567 (13)	0.2574 (3)	0.45284 (7)	0.0437 (4)	
H14	0.5295	0.1165	0.4682	0.052*	
C10	0.35198 (12)	1.6127 (3)	0.19643 (7)	0.0441 (4)	
H10	0.3955	1.7253	0.1847	0.053*	
C7	0.21950 (11)	1.2872 (3)	0.23044 (7)	0.0378 (3)	
C3	0.03236 (14)	0.4189 (4)	0.37222 (10)	0.0645 (5)	
H3A	0.0032	0.3445	0.3339	0.077*	
H3B	−0.0167	0.5349	0.3811	0.077*	
N1	0.34778 (9)	1.0947 (2)	0.29374 (6)	0.0382 (3)	
N2	0.17568 (9)	1.1066 (2)	0.25700 (6)	0.0401 (3)	
H2	0.1102	1.0698	0.2513	0.048*	
N3	0.31612 (9)	0.6757 (2)	0.36227 (6)	0.0384 (3)	
N4	0.41544 (9)	0.7421 (2)	0.36668 (6)	0.0390 (3)	
H4	0.4316	0.8680	0.3505	0.047*	
N5	0.73044 (13)	0.1896 (3)	0.49538 (7)	0.0567 (4)	
O1	0.82217 (12)	0.2456 (3)	0.49629 (8)	0.0857 (5)	
O2	0.70291 (13)	0.0185 (2)	0.52145 (7)	0.0771 (4)	
O3	0.13339 (9)	0.5214 (2)	0.36682 (6)	0.0566 (3)	
O4	0.05567 (8)	0.7645 (2)	0.29566 (6)	0.0548 (3)	
C4	0.04983 (17)	0.2514 (4)	0.42261 (11)	0.0837 (7)	
H4A	0.0745	0.3282	0.4607	0.126*	
H4B	0.1014	0.1423	0.4144	0.126*	
H4C	−0.0150	0.1746	0.4256	0.126*	

### Atomic displacement parameters (Å^2^)

**Table:**

	*U*^11^	*U*^22^	*U*^33^	*U*^12^	*U*^13^	*U*^23^
C1	0.0289 (7)	0.0419 (8)	0.0390 (8)	0.0026 (6)	0.0040 (6)	−0.0048 (6)
C12	0.0317 (7)	0.0409 (8)	0.0317 (7)	0.0023 (6)	0.0040 (6)	−0.0034 (6)
C13	0.0342 (8)	0.0451 (9)	0.0424 (8)	−0.0027 (7)	0.0063 (7)	−0.0026 (7)
C5	0.0284 (7)	0.0394 (8)	0.0388 (8)	0.0026 (6)	0.0036 (6)	−0.0068 (6)
C15	0.0431 (9)	0.0444 (9)	0.0354 (8)	0.0112 (7)	0.0012 (6)	−0.0017 (7)
C2	0.0333 (8)	0.0440 (8)	0.0465 (9)	0.0006 (7)	0.0063 (7)	−0.0017 (7)
C17	0.0349 (8)	0.0401 (8)	0.0462 (9)	−0.0013 (6)	0.0042 (7)	0.0037 (7)
C6	0.0311 (7)	0.0399 (8)	0.0382 (8)	0.0027 (6)	0.0048 (6)	−0.0064 (6)
C11	0.0305 (7)	0.0482 (9)	0.0444 (9)	−0.0024 (6)	0.0059 (6)	−0.0075 (7)
C16	0.0307 (8)	0.0520 (10)	0.0478 (9)	0.0002 (7)	0.0024 (7)	−0.0031 (8)
C8	0.0317 (8)	0.0497 (9)	0.0546 (10)	0.0042 (7)	0.0009 (7)	0.0016 (8)
C9	0.0449 (9)	0.0457 (9)	0.0504 (9)	0.0047 (7)	0.0082 (7)	0.0033 (8)
C14	0.0509 (10)	0.0395 (8)	0.0416 (8)	0.0002 (7)	0.0098 (7)	0.0019 (7)
C10	0.0420 (9)	0.0452 (9)	0.0467 (9)	−0.0055 (7)	0.0122 (7)	−0.0042 (7)
C7	0.0297 (7)	0.0396 (8)	0.0439 (8)	0.0008 (6)	0.0052 (6)	−0.0045 (7)
C3	0.0377 (9)	0.0708 (12)	0.0829 (14)	−0.0125 (9)	0.0033 (9)	0.0184 (11)
N1	0.0289 (6)	0.0420 (7)	0.0430 (7)	0.0009 (5)	0.0036 (5)	−0.0044 (6)
N2	0.0249 (6)	0.0435 (7)	0.0505 (8)	0.0004 (5)	0.0017 (5)	0.0006 (6)
N3	0.0297 (6)	0.0451 (7)	0.0402 (7)	0.0003 (5)	0.0046 (5)	−0.0049 (6)
N4	0.0279 (6)	0.0435 (7)	0.0443 (7)	−0.0005 (5)	0.0015 (5)	0.0026 (6)
N5	0.0581 (10)	0.0579 (10)	0.0501 (9)	0.0171 (8)	−0.0038 (7)	−0.0030 (8)
O1	0.0495 (8)	0.0996 (12)	0.1017 (12)	0.0226 (8)	−0.0076 (8)	0.0158 (10)
O2	0.0933 (11)	0.0556 (8)	0.0748 (10)	0.0155 (8)	−0.0109 (8)	0.0150 (7)
O3	0.0330 (6)	0.0639 (8)	0.0703 (8)	−0.0079 (5)	0.0002 (5)	0.0183 (6)
O4	0.0278 (6)	0.0640 (8)	0.0704 (8)	0.0009 (5)	0.0006 (5)	0.0136 (6)
C4	0.0629 (13)	0.0951 (17)	0.0888 (16)	−0.0217 (12)	−0.0014 (12)	0.0320 (14)

### Geometric parameters (Å, º)

**Table:**

C1—N3	1.3064 (19)	C16—H16	0.9300
C1—C5	1.457 (2)	C8—C9	1.378 (2)
C1—C2	1.490 (2)	C8—C7	1.391 (2)
C12—N4	1.3921 (19)	C8—H8	0.9300
C12—C17	1.395 (2)	C9—C10	1.404 (2)
C12—C13	1.396 (2)	C9—H9	0.9300
C13—C14	1.377 (2)	C14—H14	0.9300
C13—H13	0.9300	C10—H10	0.9300
C5—N1	1.3295 (19)	C7—N2	1.374 (2)
C5—N2	1.3659 (19)	C3—O3	1.455 (2)
C15—C16	1.381 (2)	C3—C4	1.472 (3)
C15—C14	1.381 (2)	C3—H3A	0.9700
C15—N5	1.461 (2)	C3—H3B	0.9700
C2—O4	1.2140 (18)	N2—H2	0.8600
C2—O3	1.320 (2)	N3—N4	1.3259 (17)
C17—C16	1.381 (2)	N4—H4	0.8600
C17—H17	0.9300	N5—O1	1.223 (2)
C6—N1	1.389 (2)	N5—O2	1.236 (2)
C6—C11	1.400 (2)	C4—H4A	0.9600
C6—C7	1.404 (2)	C4—H4B	0.9600
C11—C10	1.378 (2)	C4—H4C	0.9600
C11—H11	0.9300		
			
N3—C1—C5	125.50 (13)	C10—C9—H9	119.4
N3—C1—C2	114.28 (14)	C13—C14—C15	119.79 (15)
C5—C1—C2	120.20 (13)	C13—C14—H14	120.1
N4—C12—C17	118.87 (14)	C15—C14—H14	120.1
N4—C12—C13	121.10 (13)	C11—C10—C9	121.48 (15)
C17—C12—C13	120.03 (14)	C11—C10—H10	119.3
C14—C13—C12	119.47 (15)	C9—C10—H10	119.3
C14—C13—H13	120.3	N2—C7—C8	132.42 (14)
C12—C13—H13	120.3	N2—C7—C6	105.33 (13)
N1—C5—N2	112.18 (14)	C8—C7—C6	122.25 (14)
N1—C5—C1	123.37 (13)	O3—C3—C4	107.77 (15)
N2—C5—C1	124.43 (13)	O3—C3—H3A	110.2
C16—C15—C14	121.63 (14)	C4—C3—H3A	110.2
C16—C15—N5	119.41 (15)	O3—C3—H3B	110.2
C14—C15—N5	118.96 (16)	C4—C3—H3B	110.2
O4—C2—O3	123.72 (14)	H3A—C3—H3B	108.5
O4—C2—C1	123.71 (15)	C5—N1—C6	105.10 (12)
O3—C2—C1	112.57 (13)	C5—N2—C7	107.60 (12)
C16—C17—C12	120.24 (15)	C5—N2—H2	126.2
C16—C17—H17	119.9	C7—N2—H2	126.2
C12—C17—H17	119.9	C1—N3—N4	120.69 (14)
N1—C6—C11	130.57 (14)	N3—N4—C12	117.87 (13)
N1—C6—C7	109.78 (13)	N3—N4—H4	121.1
C11—C6—C7	119.65 (14)	C12—N4—H4	121.1
C10—C11—C6	118.11 (14)	O1—N5—O2	123.90 (16)
C10—C11—H11	120.9	O1—N5—C15	118.19 (17)
C6—C11—H11	120.9	O2—N5—C15	117.91 (17)
C15—C16—C17	118.83 (15)	C2—O3—C3	117.63 (13)
C15—C16—H16	120.6	C3—C4—H4A	109.5
C17—C16—H16	120.6	C3—C4—H4B	109.5
C9—C8—C7	117.22 (15)	H4A—C4—H4B	109.5
C9—C8—H8	121.4	C3—C4—H4C	109.5
C7—C8—H8	121.4	H4A—C4—H4C	109.5
C8—C9—C10	121.29 (16)	H4B—C4—H4C	109.5
C8—C9—H9	119.4		

### Hydrogen-bond geometry (Å, º)

**Table:**

*D*—H···*A*	*D*—H	H···*A*	*D*···*A*	*D*—H···*A*
N2—H2···O4^i^	0.86	2.50	3.161 (3)	134
C8—H8···O4^i^	0.93	2.54	3.258 (3)	134
N2—H2···O4	0.86	2.21	2.750 (3)	121
N4—H4···N1	0.86	2.02	2.679 (3)	133

Symmetry code: (i) −*x*, *y*+1/2, −*z*+1/2.
