# Supplementary figures and images for: Crystal structure of ethyl 2-(1H-benzimidazol-2-yl)-2-[2-(4-nitro­phen­yl)hydrazinyl­idene]acetate
Source: Acta Crystallogr E Crystallogr Commun. 2015 Mar 14;71(Pt 4):o236–7. doi: 10.1107/S2056989015004818 (PMC4438822; doi:10.1107/S2056989015004818)

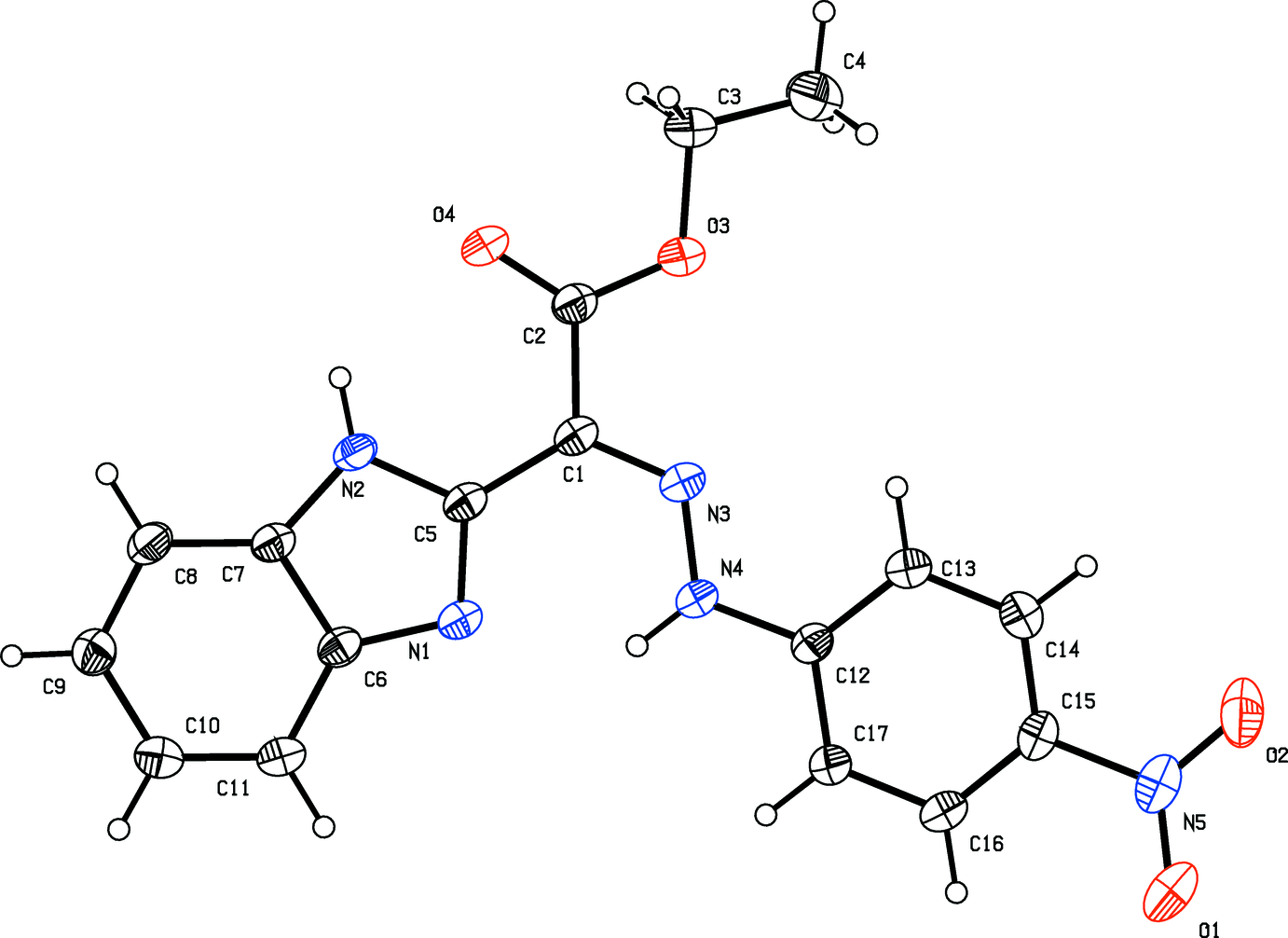

Supplement: Supplementary file 4 [file e-71-0o236-fig1.tif]

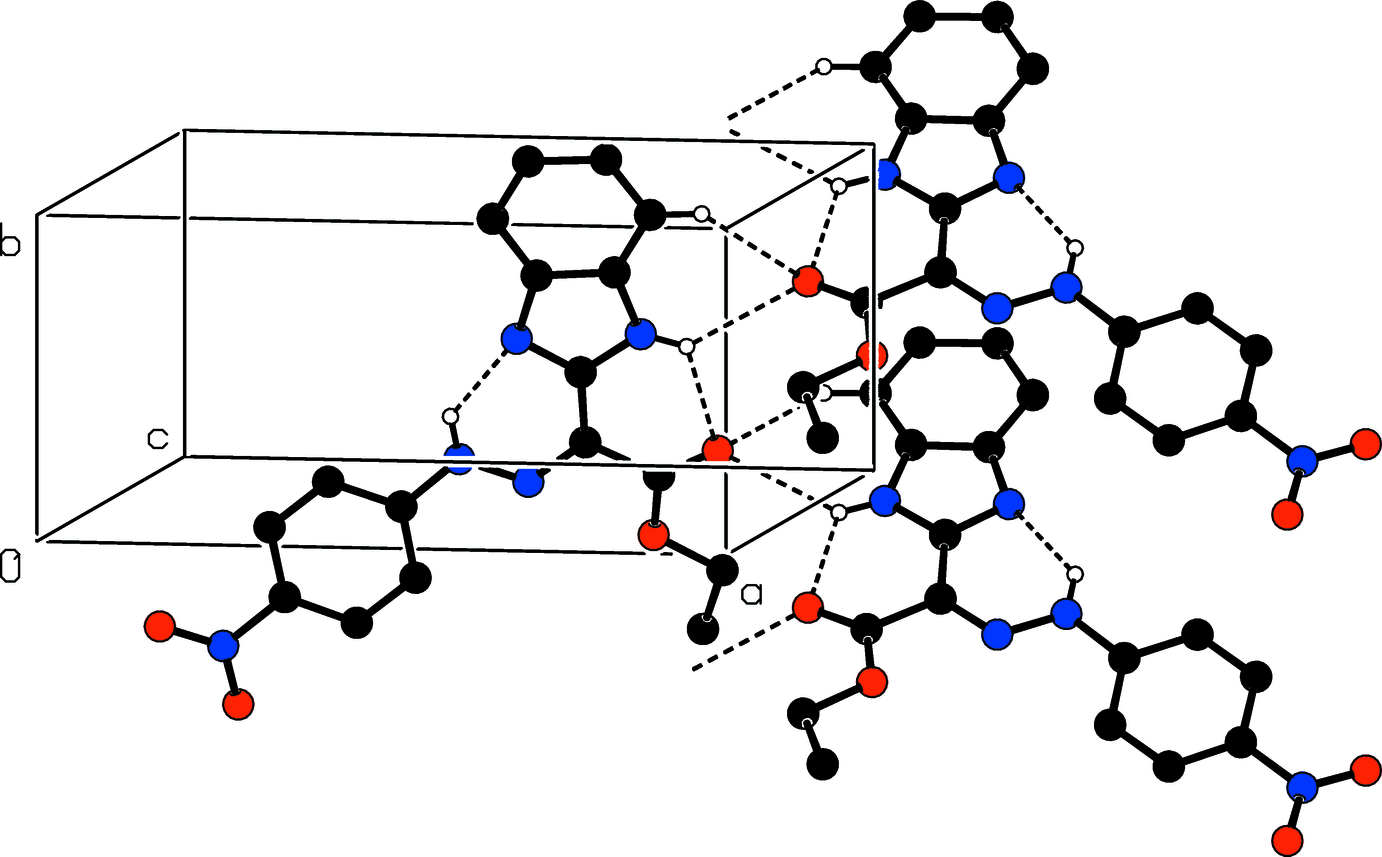

Supplement: Supplementary file 5 [file e-71-0o236-fig2.tif]
